# Application and research progress of synthetic lethality in the development of anticancer therapeutic drugs

**DOI:** 10.3389/fonc.2024.1460412

**Published:** 2024-11-25

**Authors:** Xiaoliang Gong, Chunxi Liu, Haoyang Tang, Song Wu, Qingyun Yang

**Affiliations:** State Key Laboratory of Bioactive Substance and Function of Natural Medicines, Institute of Materia Medica, Chinese Academy of Medical Sciences & Peking Union Medical College, Beijing, China

**Keywords:** synthetic lethality, precision medicine, PARP, PRMT5, WRN

## Abstract

With the tremendous success of the PARP inhibitor olaparib in clinical practice, synthetic lethality has become an important field for the discovery and development of anticancer drugs. More and more synthetic lethality targets have been discovered with the rapid development of biotechnology in recent years. Currently, many drug candidates that were designed and developed on the basis of the concept of synthetic lethality have entered clinical trials. Taking representative synthetic lethal targets Poly ADP-ribose polymerase 1 (PARP1), Werner syndrome helicase (WRN) and protein arginine methyltransferase 5 (PRMT5) as examples, this article briefly discusses the application and research progress of synthetic lethality in the development of anticancer drugs.

## 1 Introduction

Synthetic lethality refers to a phenomenon wherein simultaneous mutations in a pair of genes lead to the lethality of cells or organisms, whereas cells or organisms still survive when both genes remain wild type or when either gene is mutated (1, 2). It has become an important area of research and development for anticancer drugs: if a gene has been altered (through deletion, mutation, and amplification) in tumor cells, the use of therapeutic drugs, such as small-molecule drugs for specific regulation, especially to inhibit the expression or function of another gene, can lead to the death of tumor cells without substantially affecting normal cells. By utilizing this strategy, therapeutic drugs can specifically inhibit and/or kill tumor cells, greatly improving the therapeutic safety window of drugs.

As early as 100 years ago, geneticist Calvin Bridges observed the synthetic lethality of the genetic combinations of two mutant genes in fruit flies. In 1997, Stephen H. Friend and others published an article in *Science*, emphasizing that a mutation in a gene in tumor cells leads to changes in entire molecular signaling networks. Such changes may lead to the excessive dependence of cells on another gene and its expression product. The pharmacological inhibition of another gene or its expression product results in the specific death of tumor cells (3). These studies provided a theoretical foundation for developing anticancer drugs on the basis of synthetic lethality theory.

**Figure 1:**
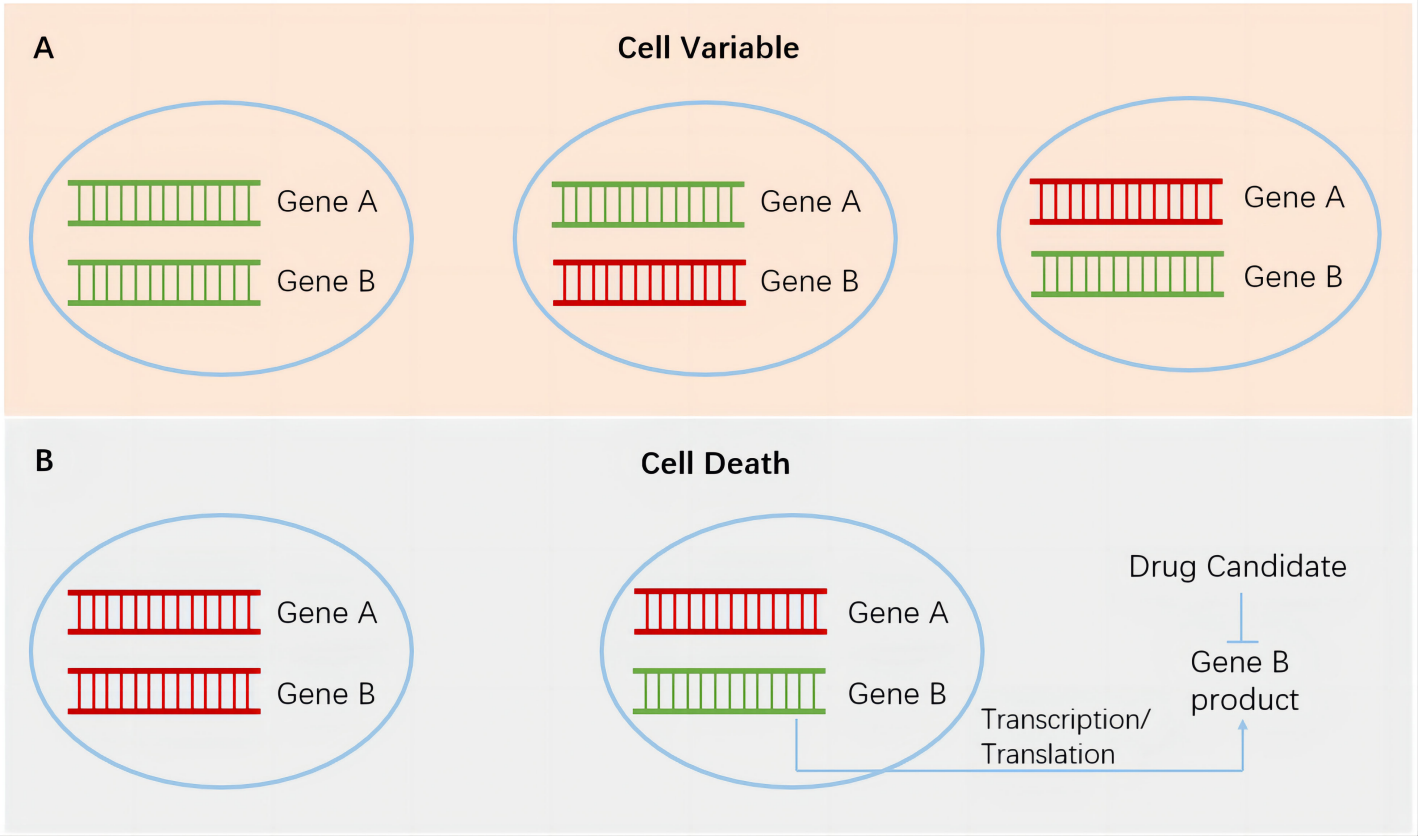
Synthetic lethality in cancer cells. Green, wild-type genes. Red, mutated genes. **(A)**, cancer cells survive when both genes remain wild type or when either gene is mutated. **(B)**, left, simultaneous mutations in a pair of genes lead to the death of cancer cells. Right, targeting gene B products in cancer cells harboring gene A mutation has the potential to achieve specific and potent cancer cell killing without or rarely affecting normal cells.

In recent years, research on synthetic lethal gene pairs has been progressing with the rapid advancement of biotechnology. The Score, Achilles, and Drive studies performed by the Sanger Institute, Broad Institute, and Novartis, respectively, utilized CRISPR-Cas9 or RNA interference technology to specifically knock out or inhibit tens of thousands of different genes in thousands of cancer cell lines harboring various genetic alterations (4–6). The dependence of cells carrying different altered genes on specific genes was studied through bioinformatics analysis. After excluding the essential genes necessary for cell survival and proliferation, a large number of synthetic lethal gene pairs were found, and a series of new targets that can be used for precision cancer treatment were identified. In each gene pair (genes A and B), using the highly variable gene A in tumor cells as a marker for tumor patient enrollment and targeting the protein product of gene B to develop specific antitumor drugs achieves specific and potent tumor cell killing without or rarely affecting normal cells (**Figure 1**). This strategy greatly improves the effectiveness and safety of drugs and provides a new direction for the development of anticancer drugs. In this article, we discuss the application and research progress of synthetic lethality-based anticancer drugs by combining those that have been marketed and those that are currently in clinical trials.

## 2 BRCA1/2 mutation and poly ADP-ribose polymerase inhibitors

Poly ADP-ribose polymerase (PARP) inhibitors are the first class of anticancer drugs developed on the basis of the concept of synthetic lethality. Currently, many PARP inhibitors have been approved for the treatment of patients harboring BRCA1/2-mutated tumors. The tremendous success of PARP inhibitors in clinical trials has led to a boom in research and development in the field of synthetic lethality. The first PARP inhibitor approved for marketing was olaparib, a PARP1/2 inhibitor, which was launched in 2014. Subsequently, rucaparib, niraparib, talazoparib, and other PARP inhibitors were successively approved for marketing, with their indications constantly expanding from ovarian cancer to other cancer types, including breast, prostate, and pancreatic cancers. At the same time, companion diagnostic biomarkers have been expanded from initial somatic and germline BRCA1/2 mutations to other DNA homologous recombination deficiencies (HRDs), and multiple assay kits have been developed or are developing for patient selection as reviewed by Murai et al. (7). The representative, commercially available assay is myChoice CDx developed by Myriad, which calculates HRD score according to loss of heterozygosity (LOH), telomeric allelic imbalance (TAI), and large-scale state transitions (LST). A score equal to or higher than 42 is defined as HRD, otherwise defined as HR-proficient. Since clinical usages of PARP inhibitors have been continuously extended from late-line treatment to front-line treatment, olaparib has become a blockbuster drug with an annual revenue of several billion dollars.

Tumor cells generally grow rapidly and DNA damage inevitably occurs during proliferation. Multiple DNA damage repair mechanisms exist in cells (8, 9). BRCA1 and BRCA2 repair DNA double-strand breaks through homologous recombination in cells, whereas PARP1 and PARP2 are key enzymes that repair DNA single-strand breaks (10, 11). BRCA1/2 mutations often occur in certain tumor types, including breast, ovarian, and prostate cancers. For patients harboring these mutations, treatments with PARP1/2 inhibitors have achieved excellent therapeutic success (12–17), resulting in several initially approved indications being approved for marketing through accelerated approval.

By identifying DNA nick marks and synthesizing poly ADP ribose (PAR) chains at the site of damage, PARP recruits repair factors and plays an important role in single strand damage repair. The inhibition of PARP activity and the inherent trapping activity of PARP inhibitors can lead to the accumulation of single chain damaged SSB and replication fork collapse, resulting in a significant increase in double chain damage. The loss of BRCA function leads to dysfunction in homologous recombination, forcing cells to utilize error prone double stranded repair mechanisms such as non-homologous end joining (NHEJ) for repair, resulting in a large production of toxic repair products, genomic instability, and ultimately cell apoptosis (18–20).

In recent years, with the continuous deepening of research on the synthetic lethality between PARP inhibitors and BRCA dysfunction, researchers have proposed modified models to explain the observed synthetic lethality phenomenon (21, 22). In short, during replication stress-induced DNA damage, BRCA has the ability to prevent the degradation of nascent DNA mediated by nucleases such as MRE11, and protect the functional integrity of the reversed fork, thereby avoiding replication fork collapse and cell apoptosis. However, PARP inhibition leads to an increase in single chain damage. Meanwhile, its inhibition of lagging strand maturation results in further growth of single strand gaps. In addition, the trapping activity of PARP inhibitors leads to longer lasting existence of DNA single stranded gaps. In BRCA deficient cells, the inability to protect the single stranded DNA gap from nuclease degradation may lead to replication catastrophe. At the same time, the single stranded DNA gap persists until mitosis and subsequent S phase, forming DSB and continuously accumulating, ultimately leading to cell death. However, the mechanism of synthetic lethality is still not fully understood and further research is needed.

Chemotherapy drugs often cause DNA double-strand damage, which provides a natural basis for combination with PARP inhibitors. The combination of PARP inhibitors and chemotherapy may even be able to overcome the strict limitations of biomarkers. However, observed hematological toxicity of PARP1/2 inhibitors has limited their further expansion in clinical applications, especially in combination with chemotherapy (10, 23) due to overlapping hematology toxicity. Although both PARP1 and PARP2 are DNA-dependent PARPs, their substrates are different. Recent studies have shown that inhibiting PARP1 alone produces a good synthetic lethal effect with BRCA mutations without consideration of blood toxicity. By contrast, inhibiting PARP2 leads to severe hematological toxicity, especially red blood cell toxicity (24, 25). All the evidences suggested that a PARP1 selective inhibitor with the absence of PARP2 binding has the potential to maintain the capability to kill BRCA-mutated tumor cells but avoid hematology toxicity. On this basis, the development of selective PARP1 inhibitors has become a research hotspot in recent years.

In search of favorable starting points for optimization and discovery of PARP1-selective inhibitors, scientists from AstraZeneca collected 7 commercial available PARP inhibitors and compared their selectivity for PARP1 (26). Among these inhibitors, FR257531 showed excellent selectivity (~300 fold) for PARP1 over PARP2, confirming the possibility of discovery of a PARP1 inhibitor. However, this molecule possesses high LogD, poor hepatic metabolism stability and cardiotoxicity risk. Scientists made much efforts to improve the druggability, leading to the discovery of AZD5305 with a selectivity of ~460 fold. The highly selective PARP1 inhibitor AZD5305 with strong trapping activity has entered the clinical trials in recent years (clinical trial no. NCT04644068 and NCT05367440). Preclinical research data showed that AZD5305 had the capability to reduce the risk of hematological toxicity while maintaining potent synthetic lethal effects (27). Importantly, in mouse CDX and PDX models covering various cancer types, AZD5305 has demonstrated efficacy and safety in combination with carboplatin (28, 29). However, despite of its high selectivity, a recent study showed that AZD5305 also exhibited a clear allosteric effect on PARP2 and retains PARP2 on DNA breaks (30). In addition, due to the unique trapping activity of PARP inhibitors, the high rate of germline mutations in BRCA mutations and functional difference between BRCA1 and BRCA2 mutations, further clinical studies are still needed to confirm whether PARP1-selective inhibitors can remarkably reduce hematological toxicity while remaining synthetic lethality with BRCA1/2 mutations (31–33).

There are currently a large number of studies on PARP inhibitor resistance, and various resistance mechanisms are summarized and discussed in Dias et al’s excellent review (34). Similar to other drugs, resistance to PARP inhibitors can be divided into two categories: intrinsic resistance and acquired resistance, including PARP1 point mutations (35), PAR glycoshydrolase (PARG) deletions (PARG can reverse PARylation and inhibit PARP activity) (36), BRCA frameshift mutations/promoter demethylation-mediated homologous recombination restoration (37), etc. To overcome drug resistance, extensive research has been conducted, such as utilizing PARG inhibitors to overcome resistance to PARP inhibitors or combining Pol-θ (involved in NHEJ double chain repair) inhibitors with PARP inhibitors (38, 39).

Researchers have long believed that the unique trapping activity of PARP inhibitors is crucial for their efficacy. Some PARP inhibitors, such as niraparib, talazoparib, and AZD5305, possess potent trapping activity. However, in clinical practice, high trapping activity does not appear to bring substantial improvement in efficacy. A recent article published by Michalis Petropoulos and others suggests that trapping activity is unnecessary for the efficacy of PARP inhibitors, but may increase safety risks such as hematology toxicity, providing new insights for the subsequent development of PARP inhibitors (40).

## 3 dMMR/MSI-H and Werner syndrome helicase inhibitors

Werner syndrome helicase (WRN) has become the hottest synthetic lethality target in recent years. In 2017, two large-scale synthetic lethality studies, Achilles and Drive, showed the strongest synthetic lethal interaction occured between WRN inhibition and dMMR/MSI-H (5, 6). Subsequent studies further confirmed the existence of potent synthetic lethality between WRN inhibition and dMMR/MSI-H (41) and conducted in-depth research on the mechanisms behind the synthetic lethal interaction.

WRN belongs to the Rec Q family of DNA helicases and is involved in multiple DNA repair pathways, which are important for maintaining genomic integrity (42). Its mutation can lead to Werner syndrome, an autosomal recessive genetic disorder characterized by premature aging and cancer susceptibility (43). In contrast to the Rec Q family members RecQ1, RecQ4, RecQ5, and BLM, WRN has two enzymatic activity centers responsible for helicase and exonuclease activities. Recent studies have shown that helicase activity, but not exonuclease activity, is crucial for the observed synthetic lethality interaction (44, 45). At the same time, this synthetic lethality effect has not been observed in other RecQ family helicases (41), indicating that WRN is the exclusive helicase with strong synthetic lethal effects with MSI-H. Further research has shown that in MSI-H tumor cells, the excessive extension of short repeat sequences, such as TA, leads to the formation of non-B DNA secondary structures, resulting in replication fork stalling, ATR activation, and WRN recruitment, which restores replication fork progression through WRN unwinding (46). WRN inhibition leads to replication fork collapse, DNA double-strand breaks, and subsequent apoptosis in MSI-H tumor cells. This study provided mechanistic insights and theoretical basis for the observed synthetic lethal effects.

MSI-H has an incidence of 10–25% in some cancer types, including colorectal, gastric, ovarian, and endometrial cancers (47). On this basis, numerous studies have been devoted to the discovery of selective WRN inhibitors. Currently, two specific WRN helicase inhibitors, HRO761 and VVD-133214, have entered the clinical trials but no clinical data has been released yet. HRO761 is a noncovalent inhibitor developed by Novartis (48), whereas VVD-133214 is a covalent inhibitor jointly developed by Vividion Therapeutics and Roche (49). Preclinical data showed that these two molecules potently inhibit MSI-H tumor cells in *in vitro* and *in vivo* models but lack a remarkable effect on normal cells and MSS tumor cells, suggesting an excellent safety window.

HRO761 binds to a non-conserved site at the interface of helicase lobes D1 and D2, and allosterically locks WRN in an inactive conformation (48) without off-targeting to other Rec Q family members. Cell panel assays confirmed excellent selectivity of HRO761 since this molecule potently inhibits most of MSI-H tumor cells at low nMs and completely spares MSS tumor cells even at concentrations of high μMs. Interestingly, in addition to helicase activity inhibition, HRO761 treatment led to significant WRN degradation though it is not a proteolysis-targeting chimera (PROTAC) molecule. Further studies suggested that PIAS4-RNF4-p97/VCP axis is critical for HRO761-induced WRN degradation (50). Different from HRO761, VVD-133214 selectively engages a cycteine C727 located in a region of the helicase domain and exhibits excellent cell growth inhibition in MSI-H tumor cells, but not in MSS cells (49).

A study performed by Picco et al. validated a positive correlation of sensitivity to WRN inhibition with expanded TA-repeats (51). This finding may be used to explain the phenomenon that some MSI-H tumor cells are not sensitive to WRN helicase inhibition since some MSI-H cells possess short TA repeats. On the other hand, to explore potential acquired resistance to WRN inhibitors, Fowler et al. continuously treated MSI-H tumor cells (HCT116 and SW48) with HRO761 and other WRN inhibitors showing a similar mechanism of action (52). Sequencing data revealed the emergence of multiple point mutations within WRN helicase domains. Subsequent SW48 xenograft model confirmed two of discovered mutations and demonstrated some new point mutations. These mutations either block inhibitor binding or affect the adoption of binding conformation. Due to distinct action mode, resistance mechanism of VVD-133214 is supposed to be different from non-covalent inhibitors such as HRO761 but no data is available yet.

Currently, there is no clinical safety data available with WRN inhibitors. In 2017, Kamath-Loeb et al. reported a specific WRN variant R834C in a population (53). These patients demonstrate some symptoms, most commonly ametropia, grey hair and some cardiovascular performance such as high blood pressure and onset of diabetes mellitus after the age of 20. Interestingly, R834C mutation leads to abolation of WRN helicase activity, but has no obvious effect on exonuclease activity. This study suggests that helicase-specific WRN inhibitors are supposed to be tolerable in clinic. Combining with high selectivity of WRN helicase inhibitors in MSI-H tumor cells over MSS normal cells, we are expecting a low safety risk in clinic and we are waiting for clinical data to verify the hypothesis.

Though immune checkpoint inhibitors have achieved dramatic success for treatment of patients haboring MSI-H/dMMR, a large population of these patients are not responsive to these treatments (54). As stated above, WRN inhibitors have shown potent anti-tumor effect in *in vitro* and *in vivo* MSI-H tumor models. WRN inhibitors have the potential to be used for the therapy of immunotherapy irresponsive and resistant MSI-H/dMMR patients. At the same time, WRN inhibitors possess excellent safety profiles and could be combined with other treatments for cancer therapy. Currently, Novartis has initiated the clinical trials to evaluate the combination of HRO761 with an anti-PD-1 antibody pembrolizumab and a chemotherapy drug irinotecan in patients harboring MSI-H and dMMR (clinical trial no. NCT05838768). As companion diagnostic methods for immune checkpoint inhibitors, the detection of MSI-H and dMMR has become increasingly mature and commercial assay kits have become widely available (47). However, there is no commercial available assay kit for measurement of TA repeat length on the market yet.

In addition to WRN inhibitors that have entered clinical trials, some molecules are currently in preclinical research stage. They include the covalent small-molecule WRN inhibitor developed by Ideaya and GSK (55), Insilico Medicine’s ISM9342 (56), and Puhe BioPharma’s PH027-1 (57). We expect that these WRN inhibitors will be clinically validated and benefit patients as soon as possible.

## 4 Methylthioadenosine phosphorylase deficiency and MAT2A/PRMT5 inhibition

The methylthioadenosine phosphorylase (MTAP) gene is located on chromosome 9p21.3 and often deleted together with CNKN2A and CDKN2B. It is the most commonly deleted gene in tumor cells, with an incidence of approximately 10% in all cancers and approximately 15% in solid tumors (58). In recent years, many studies have reported that a synthetic lethal interaction exists between the deletion of the MTAP gene and inhibition of methionine adenosyltransferase 2α (MAT2A) or protein arginine methyltransferase 5 (PRMT5) (58–60).

MAT2A is a metabolic enzyme in the body that promotes the conversion of methionine into *S*-adenosylmethionine (SAM) (61). SAM is an important methyl donor in cells and provides methyl groups for protein arginine methyltransferases (PRMTs), including PRMT5, to complete the methylation of protein substrates (62). PRMT5 promotes the symmetric dimethylation of substrate proteins, participating in RNA splicing, cell proliferation, and DNA damage repair (63). MTAP is the only enzyme in cells responsible for the degradation of methylthioadenosine (MTA). As a result, MTAP gene deletion leads to the accumulation of MTA in cells (59). Given that MTA and SAM bind to the same site on PRMT5 with similar affinity (64), the accumulation of MTA can compete with SAM for binding to PRMT5, resulting in the partial inhibition of PRMT5 activity and leading to the sensitivity of tumor cells harboring the MTAP gene deletion to the inhibition of MAT2A and PRMT5. This phenomenon is the biological basis of synthetic lethality.

As described by Marjon et al, MAT2A knockdown induced the most significant cell growth difference (log2 fold change, 15.39) in HCT116 MTAP-del and MTAP-wt isogenic cell pairs (59). In contrast, PRMT5 knockdown led to a log2 fold change of 12.62. Thus, MAT2A was ranked as top SL target for MTAP-del tumors and PRMT5 was considered as the second best. The trend was further confirmed by cell proliferation assays with small molecule inhibitors. AGI-24512, a MAT2A inhibitor, significantly inhibited cell proliferation of MTAP-del HCT116 cells (IC_50_, ~100 nM) and had no obvious impact on MTAP-wt cells (58). However, direct PRMT5 inhibition mediated by either a substrate-competitive inhibitor GSK3326595 or a SAM-competitive inhibitor JNJ64619178 did not show a clear selectivity in HCT116 isogenic cells (selectivity less than 2×) (65). Currently, several MAT2A inhibitors are undergoing clinical research, with representative molecules being Agios Therapeutics’ AG270 (66) and Ideaya’s IDE397 (67).

It is still not clear why MAT2A inhibition generates a better selectivity in isogenic cell pairs than PRMT5 inhibition. It is known that MAT2A is a ubiquitously expressed enzyme responsible for the production of SAM, and MAT2A inhibition leads to significant decrease of SAM levels (58). Since SAM is the methyl donor responsible for all intracellular transmethylation reactions, reduction of SAM production affects the activities of many methyltransferases, including PRMTs and others (68). At the same time, MTA has the capability to bind to and inhibit activity of many methyltransferases, such as PRMT5, PRMT6, PRMT3, PRMT8, EHMT1, G9a, and so on (59). Accumulated MTA in MTAP-del cells results in the second strike on activities of these enzymes following SAM reduction. Thus, MAT2A generates synthetic lethality interaction based on activity regulation of multiple methyltransferases. This is may be partly responsible for the difference of SL effects of MAT2A inhibitors and PRMT5 inhibitors.

In recent years, new-generation PRMT5 inhibitors, namely, MTA-cooperative PRMT5 inhibitors, were designed to stabilize the binding of MTA to PRMT5 further and destabilize the binding of SAM through allosteric regulation, dramatically increasing the selectivity of PRMT5 inhibitors in MTAP-deleted over wild-type cells (64). Due to specific accumulation of MTA in MTAP-del tumor cells, the new action mode make it possible to specially target tumor cells but spare normal cells with a small molecule. For example, MRTX1719, a MTA-cooperative PRMT5 inhibitor currently in Phase I clinical trial, potently inhibited cell growth of MTAP-del HCT116 tumor cells (IC_50_, 12 nM), but did not show strong inhibition on MTAP-wt HCT116 cells (IC_50_, 980 nM), generating a selectivity of ~80× (65). Since PRMT5 is essential for sustaining hematopoiesis, hematology toxicity is the major barrier to block the clinical application of first-generation non-MTA cooperated PRMT5 inhibitors (69). MTA-cooperative PRMT5 inhibitors have the ability to maintain potent anti-tumor activities, but avoid dose-limiting hematology toxicity in cancer patients harboring homozygous loss of MTAP as shown by MRTX1719 and AMG193’s clinical studies (64, 70). Both MRTX1719 and AMG193 are in phase I clinical trials and preliminary data has shown encouraging anti-tumor activities in multiple cancer types covering lung cancer, pancreatic cancer and others, and no evidence of clinically significant myelosuppression has been observed.

PRMTs are responsible for arginine methylation of protein substrates and can be divided into three different groups (68, 71). Type I PRMTs (PRMT1, 2, 3, 4, 6, 8) promotes the production of monomethylated arginine (MMA) and asymmetric dimethylarginine (ADMA), with PRMT1 responsible for over 90% ADMA. Type II PRMTs (PRMT5 and PRMT9) mediates the production of MMA and SDMA, with PRMT5 responsible for most of SDMA. Type III PRMT (PRMT5) is limited to the production of MMA only. In a study on resistance to PRMT5 inhibitors, Long et al. discovered that no significant changes in ADMA were observed in PRMT5i resistant mantle cell lymphoma cells, suggesting the possibility of type I PRMT inhibitors to overcome the resistance to PRMT5 inhibition (72). The synergistic anti-tumor effect has been confirmed in a study performed by Fedoriw et al. (73). In addition, Long et al. also showed dysregulation of multiple signaling pathways in cells resistant to PRMT5 inhibition, and highlighted the relevance of mTOR signaling with observed resistance (72).

MTA-cooperated PRMT5 inhibitors can specifically target MTAP deficient tumor cells without affecting normal cells, indicating that the precise targeting function of antibody-drug conjugate (ADC) has been achieved through small molecules, which is a breakthrough in the development of anti-tumor small molecule drugs. Currently, several MTA-cooperative PRMT5 inhibitors have entered clinical trials. They include MRTX1719 (clinical trial no. NCT05245500), AMG193 (clinical trial no. NCT05094336), TNG908 (clinical trial no. NCT05275478), TNG462 (clinical trial no. NCT05732831), and AZD3470 (clinical trial no. NCT06130553). At the same time, clinical studies have investigated the combination of MAT2A inhibitors and MTA-cooperative PRMT5 inhibitors, such as IDE397 and AMG193 (clinical trial no. NCT05975073), in patients with MTAP gene deletion.

## 5 Other synthetic lethality targets

In addition to the above synthetic lethality targets, numerous other synthetic lethality targets are currently under research. The following table lists some reported synthetic lethality targets that are not discussed in details in this article (**Table 1**).

**Table 1:** Some reported synthetic lethality targets.

Biomarker	Synthetic lethal target	Reference
BRCA1/2 mutation	Polθ	(39, 74)
BRCA1 deficiency	USP1	(75)
ATM loss of function	ATR	(76)
ERCC1 deficiency	ATR	(77)
Polθ loss	DNA-PK	(78)
TRIM37 gene amplification	PLK4	(79)
ARID1A gene mutation	EZH2	(80)
ARID1A gene mutation	EGLN1	(81)
KRAS mutation	SHP2	(82)
BRG1 deficient	BRM	(83)
RB1 loss	Aurora A	(84)
KEAP1 loss of function	ATM	(85)

## 6 Summary and prospects

Some functional correlations should exist between two targets of synthetic lethality. Fundamentally, synthetic lethality is a further expansion and application of precision medicine in clinical practice. Appropriate predictive markers offer precise guidance for the clinical use of drugs targeting functionally related targets. The emergence of synthetic lethality provides new treatment options base on numerous targets that were previously considered undruggable. Currently, with the rapid development of biotechnology, the biological mechanisms of many synthetic lethality targets have become increasingly clear, and the relevant information provides guidance and a theoretical basis for efficacy evaluation, possible drug resistance mechanisms, and drug combination options of drug candidates. At the same time, current research has shown that although the synthetic lethal effect of some targets is relatively weak, the safety window of synthetic lethality could be remarkably improved through synthetic design. For example, the synthetic lethal effect between PRMT5 inhibitors and MTAP gene deletion is weak. However, considering the context of intracellular metabolism, the safety window of synthetic lethality is greatly improved through special MTA cooperative design (64, 65). Therefore, MTA-cooperative PRMT5 inhibitors have the ability to inhibit tumor cells potently while avoid the hematological toxicity commonly caused by first-generation, non-MTA cooperative PRMT5 inhibitors, such as GSK3326595 and JNJ64619178 (64, 65).

As mentioned earlier, previous synthetic lethality studies, such as Achilles, Drive, and Score, focused mainly on tumor cells themselves. By using a large number of cell lines harboring different genetic variations and specifically inhibiting or knocking out single genes, synthetic lethality targets could be discovered through the inhibition of tumor cell growth. Therefore, the discovered synthetic lethality targets mainly focused on DNA damage repair pathways and cell cycle-related targets. These studies cannot discover targets involving the interaction between tumor cells and the microenvironment. In recent years, synthetic lethality research has gradually expanded to the fields of *in vivo* screening and tumor immunotherapy. For example, STK11 is a tumor suppressor gene that has an incidence rate of more than 10% in lung cancer and is extremely insensitive to immunotherapy (86–88). Tango Therapeutics generated a STK11-deleted MC38 mouse cancer model and, through an *in vivo* CRISPR screening platform, found that HDAC1 is a key target mediating immune evasion in STK11-deficient tumors. By using the small molecule TNG260 to inhibit the activity of HDAC1, especially its specific complex CoREST, the sensitivity of STK11-deficient tumors to PD-1 inhibitors can be remarkably enhanced. The combination of TNG260 and a PD-1 inhibitor can lead to the complete regression of tumors (89, 90). The in-depth assays showed that TNG260 treatment promoted immune cell adhesion/migration and antigen presentation, and decreased intratumoral Treg recruitment in STK11-loss tumors, leading to restoration of sensitivity to anti-PD-1 treatment (91, 92). TNG260 selectively inhibits CoREST complex of HDAC1 but not other complexes NCoR, NuRD and Sin3. In contrast, pan-HDAC inhibitor vorinostat and class I HDAC inhibitor domatinostat inhibit multiple complex forms and have no potent immune-regulation effect. Scientists from Tango therapeutics suggests that complex selectivity is critical for immune-regulation effects of HDAC1 inhibitors. We expect that with the maturation of theory and rapid development of technology in the field of synthetic lethality, additional precise therapeutic targets can be discovered in other cancer areas and even noncancer therapeutic areas, providing new options for clinical practice.

## References

[1] Setton J Zinda M Riaz N Durocher D Zimmermann M Koehler M. Synthetic lethality in cancer therapeutics: the next generation. Cancer Discov. (2021) 11:1626–35. doi: 10.1158/2159-8290.CD-20-1503 PMC8295179 33795234

[2] Huang A Garraway LA Ashworth A Weber B. Synthetic lethality as an engine for cancer drug target discovery. Nat Rev Drug Discov. (2020) 19:23–38. doi: 10.1038/s41573-019-0046-z 31712683

[3] Hartwell LH Szankasi P Roberts CJ Murray AW Friend SH. Integrating genetic approaches into the discovery of anticancer drugs. Science. (1997) 278:1064–8. doi: 10.1126/science.278.5340.1064 9353181

[4] Behan FM Iorio F Picco G Goncalves E Beaver CM Migliardi G. Prioritization of cancer therapeutic targets using crispr-cas9 screens. Nature. (2019) 568:511–6. doi: 10.1038/s41586-019-1103-9 30971826

[5] Tsherniak A Vazquez F Montgomery PG Weir BA Kryukov G Cowley GS. Defining a cancer dependency map. Cell. (2017) 170:564–76 e16. doi: 10.1016/j.cell.2017.06.010 28753430 PMC5667678

[6] McDonald ER 3rd de Weck A Schlabach MR Billy E Mavrakis KJ Hoffman GR. Project drive: A compendium of cancer dependencies and synthetic lethal relationships uncovered by large-scale, deep rnai screening. Cell. (2017) 170:577–92 e10. doi: 10.1016/j.cell.2017.07.005 28753431

[7] Murai J Pommier Y. Brcaness, homologous recombination deficiencies, and synthetic lethality. Cancer Res. (2023) 83:1173–4. doi: 10.1158/0008-5472.CAN-23-0628 37057596

[8] Wang M Chen S Ao D. Targeting DNA repair pathway in cancer: mechanisms and clinical application. MedComm (2020). (2021) 2:654–91. doi: 10.1002/mco2.103 PMC8706759 34977872

[9] Pilie PG Tang C Mills GB Yap TA. State-of-the-art strategies for targeting the DNA damage response in cancer. Nat Rev Clin Oncol. (2019) 16:81–104. doi: 10.1038/s41571-018-0114-z 30356138 PMC8327299

[10] Sonnenblick A de Azambuja E Azim HA Jr. Piccart M. An update on parp inhibitors–moving to the adjuvant setting. Nat Rev Clin Oncol. (2015) 12:27–41. doi: 10.1038/nrclinonc.2014.163 25286972

[11] Cong K Peng M Kousholt AN Lee WTC Lee S Nayak S. Replication gaps are a key determinant of parp inhibitor synthetic lethality with brca deficiency. Mol Cell. (2021) 81:3128–44 e7. doi: 10.1016/j.molcel.2021.06.011 34216544 PMC9089372

[12] Pujade-Lauraine E Ledermann JA Selle F Gebski V Penson RT Oza AM. Olaparib tablets as maintenance therapy in patients with platinum-sensitive, relapsed ovarian cancer and a brca1/2 mutation (Solo2/engot-ov21): A double-blind, randomised, placebo-controlled, phase 3 trial. Lancet Oncol. (2017) 18:1274–84. doi: 10.1016/S1470-2045(17)30469-2 28754483

[13] Moore K Colombo N Scambia G Kim BG Oaknin A Friedlander M. Maintenance olaparib in patients with newly diagnosed advanced ovarian cancer. N Engl J Med. (2018) 379:2495–505. doi: 10.1056/NEJMoa1810858 30345884

[14] Tutt ANJ Garber JE Kaufman B Viale G Fumagalli D Rastogi P. Adjuvant olaparib for patients with brca1- or brca2-mutated breast cancer. N Engl J Med. (2021) 384:2394–405. doi: 10.1056/NEJMoa2105215 PMC9126186 34081848

[15] Robson M Im SA Senkus E Xu B Domchek SM Masuda N. Olaparib for metastatic breast cancer in patients with a germline brca mutation. N Engl J Med. (2017) 377:523–33. doi: 10.1056/NEJMoa1706450 28578601

[16] de Bono J Mateo J Fizazi K Saad F Shore N Sandhu S. Olaparib for metastatic castration-resistant prostate cancer. N Engl J Med. (2020) 382:2091–102. doi: 10.1056/NEJMoa1911440 32343890

[17] Golan T Hammel P Reni M Van Cutsem E Macarulla T Hall MJ. Maintenance olaparib for germline brca-mutated metastatic pancreatic cancer. N Engl J Med. (2019) 381:317–27. doi: 10.1056/NEJMoa1903387 PMC6810605 31157963

[18] Bryant HE Schultz N Thomas HD Parker KM Flower D Lopez E. Specific killing of brca2-deficient tumours with inhibitors of poly(Adp-ribose) polymerase. Nature. (2005) 434:913–7. doi: 10.1038/nature03443 15829966

[19] Farmer H McCabe N Lord CJ Tutt AN Johnson DA Richardson TB. Targeting the DNA repair defect in brca mutant cells as a therapeutic strategy. Nature. (2005) 434:917–21. doi: 10.1038/nature03445 15829967

[20] Murai J Huang SY Das BB Renaud A Zhang Y Doroshow JH. Trapping of parp1 and parp2 by clinical parp inhibitors. Cancer Res. (2012) 72:5588–99. doi: 10.1158/0008-5472.CAN-12-2753 PMC3528345 23118055

[21] Li X Zou L. Brcaness, DNA gaps, and gain and loss of parp inhibitor-induced synthetic lethality. J Clin Invest. (2024) 134(14):e181062. doi: 10.1172/JCI181062 39007266 PMC11245158

[22] Dibitetto D Widmer CA Rottenberg S. Parpi, brca, and gaps: controversies and future research. Trends Cancer. (2024) 10:857–69. doi: 10.1016/j.trecan.2024.06.008 39004561

[23] LaFargue CJ Dal Molin GZ Sood AK Coleman RL. Exploring and comparing adverse events between parp inhibitors. Lancet Oncol. (2019) 20:e15–28. doi: 10.1016/S1470-2045(18)30786-1 PMC7292736 30614472

[24] Farres J Llacuna L Martin-Caballero J Martinez C Lozano JJ Ampurdanes C. Parp-2 sustains erythropoiesis in mice by limiting replicative stress in erythroid progenitors. Cell Death Differ. (2015) 22:1144–57. doi: 10.1038/cdd.2014.202 PMC4568570 25501596

[25] Farres J Martin-Caballero J Martinez C Lozano JJ Llacuna L Ampurdanes C. Parp-2 is required to maintain hematopoiesis following sublethal gamma-irradiation in mice. Blood. (2013) 122:44–54. doi: 10.1182/blood-2012-12-472845 23678004 PMC4918799

[26] Johannes JW Balazs A Barratt D Bista M Chuba MD Cosulich S. Discovery of 5-4-[(7-ethyl-6-oxo-5,6-dihydro-1,5-naphthyridin-3-yl)Methyl]Piperazin-1-yl-N-methylpyridine-2-carboxamide (Azd5305): A parp1-DNA trapper with high selectivity for parp1 over parp2 and other parps. J Med Chem. (2021) 64:14498–512. doi: 10.1021/acs.jmedchem.1c01012 34570508

[27] Illuzzi G Staniszewska AD Gill SJ Pike A McWilliams L Critchlow SE. Preclinical characterization of azd5305, a next-generation, highly selective parp1 inhibitor and trapper. Clin Cancer Res. (2022) 28:4724–36. doi: 10.1158/1078-0432.CCR-22-0301 PMC9623235 35929986

[28] Dellavedova G Decio A Formenti L Albertella MR Wilson J Staniszewska AD. The parp1 inhibitor azd5305 impairs ovarian adenocarcinoma progression and visceral metastases in patient-derived xenografts alone and in combination with carboplatin. Cancer Res Commun. (2023) 3:489–500. doi: 10.1158/2767-9764.CRC-22-0423 36994441 PMC10042207

[29] Herencia-Ropero A Llop-Guevara A Staniszewska AD Domenech-Vivo J Garcia-Galea E Moles-Fernandez A. The parp1 selective inhibitor saruparib (Azd5305) elicits potent and durable antitumor activity in patient-derived brca1/2-associated cancer models. Genome Med. (2024) 16:107. doi: 10.1186/s13073-024-01370-z 39187844 PMC11348616

[30] Langelier MF Lin X Zha S Pascal JM. Clinical parp inhibitors allosterically induce parp2 retention on DNA. Sci Adv. (2023) 9:eadf7175. doi: 10.1126/sciadv.adf7175 36961901 PMC10038340

[31] Hopkins TA Ainsworth WB Ellis PA Donawho CK DiGiammarino EL Panchal SC. Parp1 trapping by parp inhibitors drives cytotoxicity in both cancer cells and healthy bone marrow. Mol Cancer Res. (2019) 17:409–19. doi: 10.1158/1541-7786.MCR-18-0138 30429212

[32] Markowski MC Antonarakis ES. Brca1 versus brca2 and parp inhibitor sensitivity in prostate cancer: more different than alike? J Clin Oncol. (2020) 38:3735–9. doi: 10.1200/JCO.20.02246 PMC7655018 32870734

[33] Vendrell JA Ban IO Solassol I Audran P Cabello-Aguilar S Topart D. Differential sensitivity of germline and somatic brca variants to parp inhibitor in high-grade serous ovarian cancer. Int J Mol Sci. (2023) 24:14181. doi: 10.3390/ijms241814181 37762485 PMC10532320

[34] Dias MP Moser SC Ganesan S Jonkers J. Understanding and overcoming resistance to parp inhibitors in cancer therapy. Nat Rev Clin Oncol. (2021) 18:773–91. doi: 10.1038/s41571-021-00532-x 34285417

[35] Pettitt SJ Krastev DB Brandsma I Drean A Song F Aleksandrov R. Genome-wide and high-density crispr-cas9 screens identify point mutations in parp1 causing parp inhibitor resistance. Nat Commun. (2018) 9:1849. doi: 10.1038/s41467-018-03917-2 29748565 PMC5945626

[36] Gogola E Duarte AA de Ruiter JR Wiegant WW Schmid JA de Bruijn R. Selective loss of parg restores parylation and counteracts parp inhibitor-mediated synthetic lethality. Cancer Cell. (2018) 33:1078–93 e12. doi: 10.1016/j.ccell.2018.05.008 29894693

[37] Pettitt SJ Frankum JR Punta M Lise S Alexander J Chen Y. Clinical brca1/2 reversion analysis identifies hotspot mutations and predicted neoantigens associated with therapy resistance. Cancer Discov. (2020) 10:1475–88. doi: 10.1158/2159-8290.CD-19-1485 PMC7611203 32699032

[38] Houl JH Ye Z Brosey CA Balapiti-Modarage LPF Namjoshi S Bacolla A. Selective small molecule parg inhibitor causes replication fork stalling and cancer cell death. Nat Commun. (2019) 10:5654. doi: 10.1038/s41467-019-13508-4 31827085 PMC6906431

[39] Ceccaldi R Liu JC Amunugama R Hajdu I Primack B Petalcorin MI. Homologous-recombination-deficient tumours are dependent on poltheta-mediated repair. Nature. (2015) 518:258–62. doi: 10.1038/nature14184 PMC4415602 25642963

[40] Petropoulos M Karamichali A Rossetti GG Freudenmann A Iacovino LG Dionellis VS. Transcription-replication conflicts underlie sensitivity to parp inhibitors. Nature. (2024) 628:433–41. doi: 10.1038/s41586-024-07217-2 PMC11006605 38509368

[41] Chan EM Shibue T McFarland JM Gaeta B Ghandi M Dumont N. Wrn helicase is a synthetic lethal target in microsatellite unstable cancers. Nature. (2019) 568:551–6. doi: 10.1038/s41586-019-1102-x PMC6580861 30971823

[42] Rossi ML Ghosh AK Bohr VA. Roles of werner syndrome protein in protection of genome integrity. DNA Repair (Amst). (2010) 9:331–44. doi: 10.1016/j.dnarep.2009.12.011 PMC2827637 20075015

[43] Huang S Lee L Hanson NB Lenaerts C Hoehn H Poot M. The spectrum of wrn mutations in werner syndrome patients. Hum Mutat. (2006) 27:558–67. doi: 10.1002/humu.20337 PMC1868417 16673358

[44] Kategaya L Perumal SK Hager JH Belmont LD. Werner syndrome helicase is required for the survival of cancer cells with microsatellite instability. iScience. (2019) 13:488–97. doi: 10.1016/j.isci.2019.02.006 PMC6441948 30898619

[45] Newman JA Gavard AE Lieb S Ravichandran MC Hauer K Werni P. Structure of the helicase core of werner helicase, a key target in microsatellite instability cancers. Life Sci Alliance. (2020) 4(1):e202000795. doi: 10.26508/lsa.202000795 33199508 PMC7671478

[46] van Wietmarschen N Sridharan S Nathan WJ Tubbs A Chan EM Callen E. Repeat expansions confer wrn dependence in microsatellite-unstable cancers. Nature. (2020) 586:292–8. doi: 10.1038/s41586-020-2769-8 PMC8916167 32999459

[47] Lorenzi M Amonkar M Zhang J Mehta S Liaw K-L. Epidemiology of microsatellite instability high (Msi-H) and deficient mismatch repair (Dmmr) in solid tumors: A structured literature review. J Oncol. (2020) 2020:1807929. doi: 10.1155/2020/1807929

[48] Ferretti S Hamon J de Kanter R Scheufler C Andraos-Rey R Barbe S. Discovery of wrn inhibitor hro761 with synthetic lethality in msi cancers. Nature. (2024) 629:443–9. doi: 10.1038/s41586-024-07350-y PMC11078746 38658754

[49] Baltgalvis KA Lamb KN Symons KT Wu CC Hoffman MA Snead AN. Chemoproteomic discovery of a covalent allosteric inhibitor of wrn helicase. Nature. (2024) 629:435–42. doi: 10.1038/s41586-024-07318-y 38658751

[50] Rodriguez Perez F Natwick D Schiff L McSwiggen D Heckert A Huey M. Wrn inhibition leads to its chromatin-associated degradation via the pias4-rnf4-P97/vcp axis. Nat Commun. (2024) 15:6059. doi: 10.1038/s41467-024-50178-3 39025847 PMC11258360

[51] Picco G Rao Y Al Saedi A Lee Y Vieira SF Bhosle S. Novel wrn helicase inhibitors selectively target microsatellite-unstable cancer cells. Cancer Discov. (2024) 14:1457–75. doi: 10.1158/2159-8290.CD-24-0052 PMC7616858 38587317

[52] Fowler FC Kelly A Jeffries C Gajda J Hickson J Han F. Abstract A002: understanding mechanisms of resistance to wrn small molecule inhibitors. Mol Cancer Ther. (2024) 23:A002–A. doi: 10.1158/1538-8514.Synthleth24-a002

[53] Kamath-Loeb AS Zavala-van Rankin DG Flores-Morales J Emond MJ Sidorova JM Carnevale A. Homozygosity for the wrn helicase-inactivating variant, R834c, does not confer a werner syndrome clinical phenotype. Sci Rep. (2017) 7:44081. doi: 10.1038/srep44081 28276523 PMC5343477

[54] Oliveira AF Bretes L Furtado I. Review of pd-1/pd-L1 inhibitors in metastatic dmmr/msi-H colorectal cancer. Front Oncol. (2019) 9:396. doi: 10.3389/fonc.2019.00396 31139574 PMC6527887

[55] Rao Y Srivatsan A Liimatta M Munoz D Quirit J Shi J. Abstract 1628: A small-molecule inhibitor of wrn selectively kills msi-H cancer cells and phenocopies wrn genetic defects. Cancer Res. (2023) 83:1628–. doi: 10.1158/1538-7445.Am2023-1628

[56] Zhao P Zhu W Ding X Wan J Chen X Zhang M. Abstract 412: discovery and preclinical characterization of ism9342a, a novel, potent, and orally bioavailable wrn inhibitor that suppresses msi-H tumor growth. Cancer Res. (2024) 84:412–. doi: 10.1158/1538-7445.Am2024-412

[57] Gao F Liu B Jing L Wang J Wu Y Wan J. Abstract 5921: ph027-1, a potent and selective small-molecule wrn inhibitor that targets msi-H tumors. Cancer Res. (2024) 84:5921–. doi: 10.1158/1538-7445.Am2024-5921

[58] Kalev P Hyer ML Gross S Konteatis Z Chen CC Fletcher M. Mat2a inhibition blocks the growth of mtap-deleted cancer cells by reducing prmt5-dependent mrna splicing and inducing DNA damage. Cancer Cell. (2021) 39:209–24 e11. doi: 10.1016/j.ccell.2020.12.010 33450196

[59] Marjon K Cameron MJ Quang P Clasquin MF Mandley E Kunii K. Mtap deletions in cancer create vulnerability to targeting of the mat2a/prmt5/riok1 axis. Cell Rep. (2016) 15:574–87. doi: 10.1016/j.celrep.2016.03.043 27068473

[60] Kryukov GV Wilson FH Ruth JR Paulk J Tsherniak A Marlow SE. Mtap deletion confers enhanced dependency on the prmt5 arginine methyltransferase in cancer cells. Science. (2016) 351:1214–8. doi: 10.1126/science.aad5214 PMC4997612 26912360

[61] Maldonado LY Arsene D Mato JM Lu SC. Methionine adenosyltransferases in cancers: mechanisms of dysregulation and implications for therapy. Exp Biol Med (Maywood). (2018) 243:107–17. doi: 10.1177/1535370217740860 PMC5788143 29141455

[62] Hwang JW Cho Y Bae GU Kim SN Kim YK. Protein arginine methyltransferases: promising targets for cancer therapy. Exp Mol Med. (2021) 53:788–808. doi: 10.1038/s12276-021-00613-y 34006904 PMC8178397

[63] Kim H Ronai ZA. Prmt5 function and targeting in cancer. Cell Stress. (2020) 4:199–215. doi: 10.15698/cst2020.08.228 32743345 PMC7380451

[64] Smith CR Aranda R Bobinski TP Briere DM Burns AC Christensen JG. Fragment-based discovery of mrtx1719, a synthetic lethal inhibitor of the prmt5*Mta complex for the treatment of mtap-deleted cancers. J Med Chem. (2022) 65:1749–66. doi: 10.1021/acs.jmedchem.1c01900 35041419

[65] Engstrom LD Aranda R Waters L Moya K Bowcut V Vegar L. Mrtx1719 is an mta-cooperative prmt5 inhibitor that exhibits synthetic lethality in preclinical models and patients with mtap-deleted cancer. Cancer Discov. (2023) 13:2412–31. doi: 10.1158/2159-8290.CD-23-0669 PMC10618744 37552839

[66] Konteatis Z Travins J Gross S Marjon K Barnett A Mandley E. Discovery of ag-270, a first-in-class oral mat2a inhibitor for the treatment of tumors with homozygous mtap deletion. J Med Chem. (2021) 64:4430–49. doi: 10.1021/acs.jmedchem.0c01895 33829783

[67] Neilan CL Fischer MM Garbett D Rao AA Mandal M White MA. Abstract B027: the mat2a inhibitor ide397: A novel combination backbone for urothelial cancer subjects with mtap deficiency. Clin Cancer Res. (2024) 30:B027–B. doi: 10.1158/1557-3265.Bladder24-b027

[68] Marjon K Kalev P Marks K. Cancer dependencies: prmt5 and mat2a in mtap/P16-deleted cancers. Annu Rev Cancer Biol. (2021) 5:371–90. doi: 10.1146/annurev-cancerbio-030419-033444

[69] Liu F Cheng G Hamard PJ Greenblatt S Wang L Man N. Arginine methyltransferase prmt5 is essential for sustaining normal adult hematopoiesis. J Clin Invest. (2015) 125:3532–44. doi: 10.1172/JCI81749 PMC4588241 26258414

[70] Rodon J Prenen H Sacher A Villalona-Calero M Penel N El Helali A. First-in-human study of amg 193, an mta-cooperative prmt5 inhibitor, in patients with mtap-deleted solid tumors: results from phase I dose exploration. Ann Oncol. (2024) in press. doi: 10.1016/j.annonc.2024.08.2339 39293516

[71] Wu Q Schapira M Arrowsmith CH Barsyte-Lovejoy D. Protein arginine methylation: from enigmatic functions to therapeutic targeting. Nat Rev Drug Discov. (2021) 20:509–30. doi: 10.1038/s41573-021-00159-8 33742187

[72] Long ME Koirala S Sloan S Brown-Burke F Weigel C Villagomez L. Resistance to prmt5-targeted therapy in mantle cell lymphoma. Blood Adv. (2024) 8:150–63. doi: 10.1182/bloodadvances.2023010554 PMC10787272 37782774

[73] Fedoriw A Rajapurkar SR O'Brien S Gerhart SV Mitchell LH Adams ND. Anti-tumor activity of the type I prmt inhibitor, gsk3368715, synergizes with prmt5 inhibition through mtap loss. Cancer Cell. (2019) 36:100–14 e25. doi: 10.1016/j.ccell.2019.05.014 31257072

[74] Zatreanu D Robinson HMR Alkhatib O Boursier M Finch H Geo L. Poltheta inhibitors elicit brca-gene synthetic lethality and target parp inhibitor resistance. Nat Commun. (2021) 12:3636. doi: 10.1038/s41467-021-23463-8 34140467 PMC8211653

[75] Lim KS Li H Roberts EA Gaudiano EF Clairmont C Sambel LA. Usp1 is required for replication fork protection in brca1-deficient tumors. Mol Cell. (2018) 72:925–41 e4. doi: 10.1016/j.molcel.2018.10.045 30576655 PMC6390489

[76] Menezes DL Holt J Tang Y Feng J Barsanti P Pan Y. A synthetic lethal screen reveals enhanced sensitivity to atr inhibitor treatment in mantle cell lymphoma with atm loss-of-function. Mol Cancer Res. (2015) 13:120–9. doi: 10.1158/1541-7786.MCR-14-0240 25232030

[77] Mohni KN Kavanaugh GM Cortez D. Atr pathway inhibition is synthetically lethal in cancer cells with ercc1 deficiency. Cancer Res. (2014) 74:2835–45. doi: 10.1158/0008-5472.CAN-13-3229 PMC4043842 24662920

[78] Patterson-Fortin J Bose A Tsai WC Grochala C Nguyen H Zhou J. Targeting DNA repair with combined inhibition of nhej and mmej induces synthetic lethality in tp53-mutant cancers. Cancer Res. (2022) 82:3815–29. doi: 10.1158/0008-5472.CAN-22-1124 PMC9588747 35972384

[79] Meitinger F Ohta M Lee KY Watanabe S Davis RL Anzola JV. Trim37 controls cancer-specific vulnerability to plk4 inhibition. Nature. (2020) 585:440–6. doi: 10.1038/s41586-020-2710-1 PMC7501188 32908304

[80] Bitler BG Aird KM Garipov A Li H Amatangelo M Kossenkov AV. Synthetic lethality by targeting ezh2 methyltransferase activity in arid1a-mutated cancers. Nat Med. (2015) 21:231–8. doi: 10.1038/nm.3799 PMC4352133 25686104

[81] Briggs KJ Min C Zhang H Huang A. Abstract 2892: egln1 is a synthetic lethal target in arid1a-mutant ovarian cancer. Cancer Res. (2018) 78:2892–. doi: 10.1158/1538-7445.Am2018-2892

[82] Mainardi S Mulero-Sanchez A Prahallad A Germano G Bosma A Krimpenfort P. Shp2 is required for growth of kras-mutant non-small-cell lung cancer in vivo. Nat Med. (2018) 24:961–7. doi: 10.1038/s41591-018-0023-9 29808006

[83] Hoffman GR Rahal R Buxton F Xiang K McAllister G Frias E. Functional epigenetics approach identifies brm/smarca2 as a critical synthetic lethal target in brg1-deficient cancers. Proc Natl Acad Sci USA. (2014) 111:3128–33. doi: 10.1073/pnas.1316793111 PMC3939885 24520176

[84] Gong X Du J Parsons SH Merzoug FF Webster Y Iversen PW. Aurora a kinase inhibition is synthetic lethal with loss of the rb1 tumor suppressor gene. Cancer Discov. (2019) 9:248–63. doi: 10.1158/2159-8290.CD-18-0469 30373917

[85] Li H Liu Y Xiao Y Wilson CN Bai HJ Jones MD. Crispr metabolic screen identifies atm and keap1 as targetable genetic vulnerabilities in solid tumors. Proc Natl Acad Sci USA. (2023) 120:e2212072120. doi: 10.1073/pnas.2212072120 36724254 PMC9963842

[86] Koyama S Akbay EA Li YY Aref AR Skoulidis F Herter-Sprie GS. Stk11/lkb1 deficiency promotes neutrophil recruitment and proinflammatory cytokine production to suppress T-cell activity in the lung tumor microenvironment. Cancer Res. (2016) 76:999–1008. doi: 10.1158/0008-5472.CAN-15-1439 26833127 PMC4775354

[87] Shang X Li Z Sun J Zhao C Lin J Wang H. Survival analysis for non-squamous nsclc patients harbored stk11 or keap1 mutation receiving atezolizumab. Lung Cancer. (2021) 154:105–12. doi: 10.1016/j.lungcan.2021.02.010 33640623

[88] Guaitoli G Tiseo M Di Maio M Friboulet L Facchinetti F. Immune checkpoint inhibitors in oncogene-addicted non-small cell lung cancer: A systematic review and meta-analysis. Transl Lung Cancer Res. (2021) 10:2890–916. doi: 10.21037/tlcr-20-941 PMC8264334 34295687

[89] Patel A Sahu S Geng K Punekar S Stephens J Deng J. Abstract 3916: tng260, a small molecule corest inhibitor, sensitizes stk11-mutant nsclc to anti-pd1 immunotherapy. Cancer Res. (2024) 84:3916–. doi: 10.1158/1538-7445.Am2024-3916 PMC12521918 40882030

[90] Ahronian LG Zhang M Min C Tsai AW Ermolieff J McCarren P. Abstract nd12: tng260: A novel, orally active, corest-selective deacetylase inhibitor for the treatment of stk11-mutant cancers. Cancer Res. (2023) 83:ND12–ND. doi: 10.1158/1538-7445.Am2023-nd12

[91] Ahronian L Zhang M Shahagadkar P Bejnood A Tsai AW Min C. 870 corest inhibition by tng260 increases expression of immunomodulatory genes in stk11-mutant cancer and sensitizes to immune checkpoint blockade. J Immunother Cancer. (2023) 11:A969–A. doi: 10.1136/jitc-2023-SITC2023.0870

[92] Ahronian L Wu X Zhang M Min C Tsai A Ermolieff J. 444 tng260, a corest-selective deacetylase inhibitor, reverses anti-pd1 resistance driven by loss of stk11. J Immunother Cancer. (2022) 10:A464–A. doi: 10.1136/jitc-2022-SITC2022.0444

